# Efficacy of single-dose intravenous immunoglobulin administration for severe sepsis and septic shock

**DOI:** 10.1186/2052-0492-1-4

**Published:** 2013-10-23

**Authors:** Nobuyuki Hamano, Kenichiro Nishi, Aki Onose, Akihisa Okamoto, Takeshi Umegaki, Etsuko Yamazaki, Kiichi Hirota, Hiroe Ookura, Hakuo Takahashi, Koh Shingu

**Affiliations:** General Intensive Care Unit, Hirakata Hospital, Kansai Medical University, 2-3-1 Shinmachi, Hirakata, Osaka, 573-1191 Japan; Division of Anesthesiology, National Center for Global Health and Medicine, 1-21-1 Toyama, Shinjuku-ku, Tokyo, 162-8655 Japan; Department of Anesthesiology, Hirakata Hospital, Kansai Medical University, 2-3-1 Shinmachi, Hirakata, Osaka, 573-1191 Japan; Department of Clinical Laboratory, Hirakata Hospital, Kansai Medical University, 2-3-1 Shinmachi, Hirakata, Osaka, 573-1191 Japan; Department of Clinical Sciences and Laboratory Medicine, Hirakata Hospital, Kansai Medical University, 2-3-1 Shinmachi, Hirakata, Osaka, 573-1191 Japan

**Keywords:** Severe sepsis, Intravenous immunoglobulins, Single-dose administration

## Abstract

**Background:**

Although some studies conducted outside of Japan have addressed the effectiveness of intravenous immunoglobulins (IVIG) in treating infections, the dosing regimens and amounts used in Japan are very different from those reported. Here, we investigate the effectiveness of single-dose administration of IVIG in sepsis patients in Japan.

**Methods:**

We analyzed 79 patients admitted to the intensive care unit (ICU) of a tertiary care institution due to severe sepsis or septic shock. Patients were randomly divided into a group that was administered standard divided doses of IVIG (5 g/day for 3 days, designated the S group) or a group that was administered a standard single dose of IVIG (15 g/day for 1 day, H group); freeze-dried sulfonated human IVIG was used. The longitudinal assessment of procalcitonin (PCT) levels, C-reactive protein (CRP) levels, white blood cell count, blood lactate levels, IL-6 levels, Sequential Organ Failure Assessment (SOFA) score, and Systemic Inflammatory Response Syndrome (SIRS) was conducted. We also assessed mechanical ventilation duration (days), ICU stay (days), 28-day survival rate, and 90-day survival rate.

****Results**:**

The study showed no significant differences in PCT levels, CRP levels, 28-day survival rate, and 90-day survival rate between the two groups. However, patients in the H group showed improvements in the various SIRS diagnostic criteria, IL-6 levels, and blood lactate levels in the early stages after IVIG administration. In light of the non-recommendation of IVIG therapy in the Surviving Sepsis Campaign Guidelines 2012, our findings of significant early post-administration improvements are noteworthy. IVIG's anti-inflammatory effects may account for the early reduction in IL-6 levels after treatment, and the accompanying improvements in microcirculation may improve blood lactate levels and reduce SOFA scores. However, the low dosages of IVIG in Japan may limit the anti-cytokine effects of this treatment. Further studies are needed to determine appropriate treatment regimens of single-dose IVIG.

**Conclusions:**

In this study, we investigated the effectiveness of single-dose IVIG treatment in patients with severe sepsis or septic shock. Although there were no significant effects on patient prognoses, patients who were administered single-dose IVIG showed significantly improved IL-6 levels, blood lactate levels, and disease severity scores.

## Background

Sepsis refers to a set of syndromes that occur as a result of a systemic inflammatory response to an infection. Without appropriate treatment, there is a high incidence of supervening disseminated intravascular coagulation (DIC) or multiple organ failure (MOF) [1], leading to extremely poor outcomes for patients.

In 2004, the Surviving Sepsis Campaign Guidelines (SSCG), a set of comprehensive management guidelines for the treatment of sepsis, were published [2]; the most recent revision—SSCG 2012—was published in 2013 [3]. These guidelines contain details on initial resuscitation, antimicrobial therapy, and infection source control. Although the use of intravenous immunoglobulins (IVIG) has been approved for the treatment of immune diseases such as idiopathic thrombocytopenic purpura and Kawasaki disease, its use in severe infections or sepsis has yet to be approved by the US Food and Drug Administration (FDA) and has also been discouraged in SSCG 2012 as a treatment for adult sepsis patients. However, IVIG has been shown to be effective in treating severe infections [4, 5], and several reviews have demonstrated the efficacy of IVIG as a supplemental drug for treating sepsis [6–9]. In the early stage of sepsis, low concentration of immunoglobulins is predicted because IgG production in patients with primary infection needs 1–2 weeks, and lots of IgG are burned up for inhibiting bacteria and toxin in secondary infection. Therefore, it is expected that IVIG administration for treatment of sepsis is effective [10]. Furthermore, there are reports [11, 12] that a single dose of IVIG for Kawasaki disease and idiopathic thrombocytopenic purpura is more effective than divided doses. In Japan, IVIG administration is approved for severe infection, but the standard dosages and administration (5 g/day for 3 days) are far below the amount on several reports set forth above. Therefore, we tested the hypothesis that a single-dose IVIG treatment in patients with severe sepsis and septic shock in Japan is more effective than divided doses.

## Methods

This study was approved by an internal ethics committee at its inception. In addition, the purpose of the study was explained to candidate research subjects or their legal representatives either verbally or in writing, and written informed consent was obtained for each participant before their inclusion in the study.

### Study sample

The study sample comprised patients who were admitted to the intensive care unit (ICU) of a tertiary care urban hospital between July 2009 and August 2012, and fulfilled the diagnostic criteria for severe sepsis or septic shock [13]. All study participants were immediately started on early goal-directed therapy [14] after diagnosis of severe sepsis or septic shock, using therapeutic strategies compliant with SSCG 2008 [15]. Patients were excluded from analysis if they fulfilled the following criteria: patients aged 17 years or younger, patients with existing allergies to IVIG or antibiotics, patients with severe liver dysfunction or kidney disease (except for organ dysfunctions that can be caused by sepsis), patients with malignant neoplasms, patients in an immunodeficient state, patients who had acute myocardial infarctions or heart failures within 6 weeks prior to the index admission, patients (or their legal representatives) who did not consent to participating in the study, and patients deemed inappropriate for the study by the principal investigating clinician.

Research subjects were randomly allocated into two groups: a standard divided IVIG dose (5 g/day for 3 days) group designated as the S group and a standard single dose (15 g/day for 1 day) group designated as the H group. These regimens were based on dosages and administration approved for severe infection in Japan.

In both groups, treatment using freeze-dried sulfonated human IVIG was initialized within 24 h of diagnosing sepsis. Patient characteristics were evaluated using age; gender; height; weight; Acute Physiology and Chronic Health Evaluation (APACHE) II score; APACHE II score excluding Glasgow Coma Scale (GCS), APACHE(−GCS); Sequential Organ Failure Assessment (SOFA) score; SOFA score excluding GCS, SOFA(−GCS); disease severity classification; and underlying disease that led to sepsis.

The various values of the following diagnostic criteria were longitudinally assessed throughout each patient's hospitalization episode: procalcitonin (PCT) levels, C-reactive protein (CRP) levels, white blood cell (WBC) count, blood lactate levels, SOFA score, SOFA(−GCS) score, and Systemic Inflammatory Response Syndrome (SIRS).

**Figure 1:**
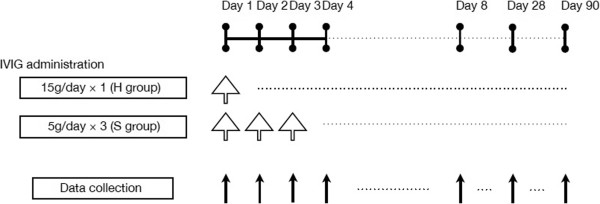
**Schedule of IVIG administration and clinical data collection time points.** White arrows indicate IVIG administration, whereas black arrows indicate data collection. IVIG, intravenous immunoglobulin.

In addition, we also analyzed the following outcome measures to evaluate patient prognosis: mechanical ventilation duration (in days), ICU stay duration (in days), 28-day survival rate, and 90-day survival rate. With the day of initial IVIG administration designated as day 1, the longitudinal assessments of patient baseline and clinical characteristics were conducted on days 1, 2, 3, 4, and 8 (Figure 1). Various parameters for day 1 were assessed on ICU admission, and assessments for days 2 and 3 of the S group were conducted prior to IVIG administration for that day. Also, plasma IL-6 was extracted and cryopreserved at the various time points, and IL-6 plasma concentration was measured using chemiluminescent enzyme immunoassay (Human IL-6 CLEIA, Fujirebio Corp., Tokyo, Japan).

Many of the research subjects in this study had undergone mechanical ventilation or had been administered sedatives, but assessments of the central nervous system in such patients have been reported to be heavily dependent on the individual assessor [16]. Therefore, we have included APACHE II scores and SOFA scores that omit GCS assessment, which measures the level of patient consciousness. In addition, APACHE II scores should be evaluated by using the worst value in the first 24 h, under normal circumstances. However, we evaluated APACHE II scores by the worst value from ICU admission to initial IVIG administration, as IVIG should be administered as soon as possible for treating sepsis.

### Statistical analysis

For assessments of patient characteristics between the two groups, we used the unpaired *t* test, Mann-Whitney *U* test, chi-square test for independence, and Fisher's exact test as appropriate for the data type for each variable. Transitions in clinical data were assessed using unpaired *t* tests. Changes in the various time points within each of the two groups were also analyzed using one-way analysis of variance (ANOVA), followed by a multiple comparison using Scheffe's method. Continuous variables were presented as mean values ± standard error of the mean (SEM), whereas ordinal variables were presented as median values (interquartile range (IQR)).

Patient prognoses were measured using Kaplan-Meier survival curves and the log-rank test. Statistical significance was set at *P* < 0.05. All statistical analyses were conducted using JMP, version 10.0 (SAS Institute Inc., Cary, NC, USA).

## Results

### Patient characteristics

Patient characteristics are presented in Table 1. The 79 research subjects in the study had a mean age of 67.2 ± 1.5 years, and the 28-day and 90-day survival rates were 86.1% and 78.5%, respectively. The APACHE II score was 27 (IQR 8–42), and the SOFA score was 10 (IQR 3–17). The most frequent source of infection for both groups was generalized peritonitis. The H group had a slightly higher proportion of men, whereas the S group had more patients presenting with urinary tract infections. However, there were no differences between the two groups with respect to the proportion of patients with severe sepsis or septic shock, and no statistically significant differences in patient characteristics were observed.

**Table 1:** **Patient characteristics (** ***n*** **= 79)**

	S group (***n*** = 42)^a^	H group (***n*** = 37)^a^	***P*** value
Age (years)	66.7 ± 2.0	67.7 ± 2.2	0.744
Gender (male/female)	21/21	25/12	0.057
Body height (cm)	159.9 ± 1.4	161.4 ± 1.6	0.465
Body weight (kg)	54.6 ± 2.0	53.5 ± 2.0	0.699
APACHE II score	25.5 (13–39)	27 (8–43)	0.115
APACHE II(−GCS)	17 (8–29)	17 (8–32)	0.936
SOFA score	11 (3–15)	10 (4–17)	0.462
SOFA(−GCS)	8 (2–14)	6 (1–13)	0.117
Clinical stratification (sever sepsis/septic shock)	4/38	5/32	0.418
Source of infection			
Generalized peritonitis	15 (35.7%)	13 (35.1%)	
Pneumonia	4 (9.5%)	6 (16.2%)	
Urinary tract	6 (14.3%)	2 (5.4%)	
Bacteremia	4 (9.5%)	3 (8.1%)	
Cellulitis	2 (4.8%)	2 (5.4%)	
Biliary tract	2 (4.8%)	1 (2.7%)	
Others	5 (11.9%)	6 (16.2%)	
Unknown	4 (9.5%)	4 (10.8%)	

^a^Patients in the S group were administered a standard IVIG dose of 5 g/day for 3 days; patients in the H group were administered a single high dose of 15 g/day for 1 day. Continuous variables: mean ± SEM; ordinal variables: median (interquartile); nominal variables: percentage. APACHE II, Acute Physiology and Chronic Health Evaluation II; APACHE II(−GCS), APACHE II without Glasgow Coma Scale; SOFA, Sequential Organ Failure Assessment; SOFA(−GCS), SOFA without Glasgow Coma Scale.

### Temporal transitions in clinical data

**Figure 2:**
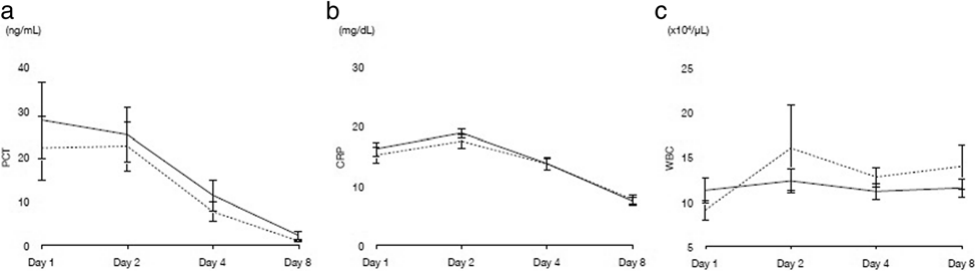
**Time course results of laboratory data. (a)** PCT levels, **(b)** CRP levels, and **(c)** WBC count. Solid lines indicate the H group, and dashed lines indicate the S group.

**Figure 3:**
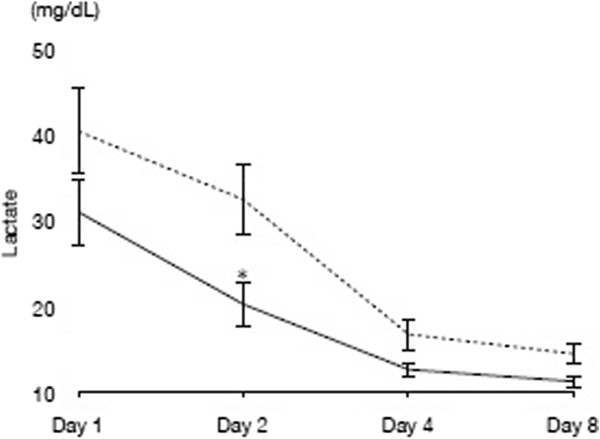
**Time course results of blood lactate levels.** *P < 0.05 when compared with the S group. Solid line indicates the H group, and dashed line indicates the S group.

**Figure 4:**
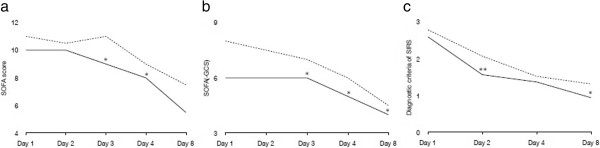
**Time course results of (a) SOFA score, (b) SOFA(−GCS), and (c) SIRS criteria.** *P < 0.05, **P < 0.01 when compared with the S group. Solid lines indicate the H group, and dashed lines indicate the S group. SOFA, Sequential Organ Failure Assessment; GCS, Glasgow Coma Scale; SIRS, Systemic Inflammatory Response Syndrome.

There were no significant differences in temporal transitions in PCT values observed between the two groups (Figure 2a). Similar results were observed for CRP values and WBC count (Figure 2b,c). There was a statistically significant difference in blood lactate levels on day 2 between both groups (*P* = 0.02), as shown in Figure 3. The H group was found to have significantly lower SOFA and SOFA(−GCS) scores in the early stages after IVIG administration (SOFA score for day 3: *P* = 0.04; SOFA(−GCS) score for day 3: *P* = 0.04), with differences continuing for the first week (Figure 4a,b). With regard to the various diagnostic criteria for SIRS, we also observed significantly lower scores (day 2: *P* < 0.01) in the H group in the early stages after IVIG administration (Figure 4c).

**Figure 5:**
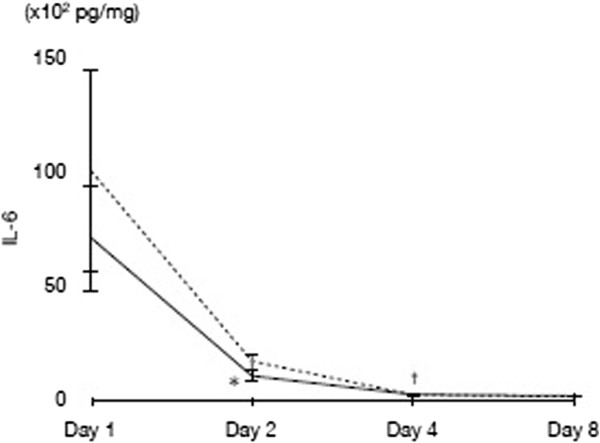
**Time course of IL-6 levels.** *P < 0.01 compared with day 1 (H group only); ^†^ *P* < 0.01 compared with day 2 (both groups), using one-way ANOVA, followed by a multiple comparison using Scheffe's method. Solid line indicates the H group, and dashed line indicates the S group. IL-6, Interleukin-6.

In addition, the results revealed a general tendency for temporal reductions in IL-6 levels in the H group across the time points, although this tendency was not found to have overall statistical significance. However, Figure 5 shows a statistically significant reduction (*P* < 0.01) in IL-6 levels in the H group between day 1 and day 2, whereas this was not observed in the S group. For the difference between day 2 and day 3, both groups showed a significant reduction in IL-6 levels.

### Clinical course

There were no significant differences in mechanical ventilation duration between the two groups or in the proportions of patients who had undergone tracheal intubation. Additionally, there were also no significant differences in ICU stay duration.

### Patient prognosis

Kaplan-Meier survival curves and the log-rank test demonstrated that the H group tended to have higher, albeit non-significant, 28-day survival rates and 90-day survival rates when compared with the S group (not shown in the figure).

## Discussion

Despite dramatic progress in treatment modalities, mortality rates for patients with serious sepsis remain high [17, 18], and a meta-analysis conducted by Friedman et al. [19] has shown the mortality rate associated with septic shock patients to be 49.7%. Factors such as the overall increases in patient age, advances in drug therapies, increasingly complex surgical procedures, and the emergence of multiple drug-resistant bacteria are thought to contribute to the continued increase in the number of patients with sepsis [20]. There is, therefore, an urgent need to further advance treatment modalities and adjunctive therapies.

In SSCG 2008 [15], the administration of IVIG was recommended in children due to a study [21] that demonstrated reduced mortality as a result of this treatment. However, this recommendation did not extend to include adult patients. Subsequently, the recommendation for IVIG administration was rescinded in the SSCG 2012 [3] due to studies such as the large-scale multi-institutional collaborative SBITS study involving 624 adult patients [22] and an investigation of 3,493 children [23]. In contrast, while the Japanese Guidelines for the Management of Sepsis [24] have not acknowledged the ameliorative effects of IVIG treatment on mortality rates, they have recognized that its use may be considered in the early stages of therapy as a means to reduce mechanical ventilation duration and improve survival rates in the ICU [22].

The regimen and dosage of IVIG in Japan are substantially lower than in many other countries. A study conducted in Japan in 2000 [25] analyzed the effects when patients with infections who showed no improvement in symptoms after 3 days of antibiotic therapy were administered IVIG (5 g/day for 3 days). When compared with similar patients who were not administered IVIG, the IVIG group showed significant improvements in disease symptoms. Although the negative conversion rate of the causative organism and the CRP rate of change were not significantly different between the two groups, a composite score comprised of fever and other symptoms was developed to measure the effectiveness of the therapy, which showed that the IVIG group had significantly higher effectiveness. However, the definition for severe sepsis was unclear, and the outcome measures used (reductions in fever and improvements in symptoms) were relatively ambiguous. Therefore, the utilization standards, regimen, and dosages reported in this study may be inconclusive.

Accordingly, we have conducted an investigation of the effectiveness of single-dose IVIG in patients with severe sepsis or septic shock. In this randomized controlled study, we did not observe significant differences between the two groups in CRP levels (a standard infection marker), PCT levels (which are thought to reflect the severity of sepsis) [26, 27], ICU stay duration, and patient prognoses. However, when compared with the standard divided doses IVIG group, the standard single-dose IVIG group was shown to have better results in the early post-administration stages for factors such as IL-6 levels, blood lactate levels, and SOFA score.

The mechanism of action for IVIG against infections has been reported to include the stimulation of Fc receptor-mediated antibiotic-dependent cellular cytotoxicity, neutralization of toxins and viruses, suppression of cytokine activity, promotion of complement-mediated bacteriolysis, and opsonization of targets to promote phagocytosis [28]. As some aspects of the mechanism still remain unclear, it would not be possible to conclusively explain the findings of this study. However, the fact [29] that IVIG formulations include anti-IL-6 antibodies may account for the early reduction in IL-6 levels after treatment, and the accompanying improvements in microcirculation may ostensibly improve blood lactate levels and reduce the SOFA score. A multi-institutional study has shown that improvements to blood lactate levels are associated with reductions in mortality risk [30], and these findings resulted in the reestablishment of the normalization of blood lactate levels at a recommended measure in SSCG 2012 [3]. From this perspective, the single-dose administration of IVIG could therefore also be thought of as being an effective treatment. In this study, we did not observe any adverse events occurring as a result of IVIG administration such as anaphylactoid symptoms or thrombocytopenia.

There were several limitations in this study. First, there may be a large degree of variation in the time to treatment among the research subjects. Next, the dosage of IVIG was not based on subject body weight but was standardized for all subjects. Also, we did not account for the anti-cytokine effects from other treatments such as blood purification therapy, steroids, sivelestat, and recombinant thrombomodulin. Additionally, there is a possibility that the pathology of sepsis differs according to the underlying illnesses. Finally, the levels of IgG in the blood were not assessed prior to administering IVIG, and there may therefore be a large degree of variation in these levels among the subjects.

A minimum of 2.0 g/kg body weight of immunoglobulins has been reported to be necessary in order to sufficiently neutralize the rise of IL-6 levels due to sepsis [31], which would be difficult to achieve in Japan given the reduced anti-cytokine effects from the lower immunoglobulin dosages. More studies are needed to further understand the costs, risk of infection, mechanism of utility, appropriate dosages, and modes of administration of single-dose IVIG treatment.

## Conclusions

In this study, we shed light on the effectiveness of single-dose IVIG treatment in patients with severe sepsis or septic shock in Japan. Although the study did not show any significant effects on patient prognoses, subjects who were administered a standard single dose of IVIG showed significantly improved early-stage IL-6 levels, blood lactate levels, and disease severity scores when compared with patients administered with standard divided doses.
